# Extended Spectrum Beta-Lactamase (ESBL) Produced by Gram-Negative Bacteria in Trinidad and Tobago

**DOI:** 10.1155/2021/5582755

**Published:** 2021-08-23

**Authors:** Patrick Eberechi Akpaka, Angel Vaillant, Clyde Wilson, Padman Jayaratne

**Affiliations:** ^1^Department of Para-Clinical Sciences, Pathology and Microbiology Unit, Faculty of Medical Sciences, The University of the West Indies, St. Augustine, Trinidad and Tobago; ^2^Department of Pathology and Microbiology, King Edward VII Memorial Hospital, Hamilton, Bermuda; ^3^Department of Pathology and Molecular Medicine Sciences, McMaster University, Hamilton, Ontario, Canada

## Abstract

Gram-negative bacterial infections are a global health problem. The production of beta-lactamase is still the most vital factor leading to beta-lactam resistance. In Trinidad and Tobago, extended spectrum beta-lactamase (ESBL) production has been detected and reported mainly in the isolates of *Klebsiella pneumoniae* and *Escherichia coli* and constitutes a public health emergency that causes high morbidity and mortality in some patients. In this literature review, the authors cover vast information on ESBL frequency and laboratory detection using both conventional and molecular methods from clinical data. The aim is to make the reader reflect on how the actual knowledge can be used for rapid detection and understanding of the spread of antimicrobial resistance problems stemming from ESBL production among common Gram-negative organisms in the health care system.

## 1. Introduction

Gram-negative bacterial infections are a global health problem. The production of beta-lactamase is still the most vital factor leading to beta-lactam resistance. The dissemination of extended spectrum beta-lactamase (ESBL) production is alarming. In a study conducted by Leylabadlo et al., ESBL production occurred most frequently in *Escherichia coli* and *Klebsiella pneumoniae*; however, other *Enterobacteriaceae* family members have also been identified to produce these enzymes [1, 2]. More than 200 ESBLs have now been characterized, and these are detailed on the authoritative website on the nomenclature of ESBLs hosted by George Jacoby and Karen Bush (http://www.lahey.org/studies/webt.htm). Over 30 different countries have published research on ESBLs, reflecting the truly worldwide distribution of ESBL-producing organisms. In Trinidad and Tobago, several ESBL-producing Gram-negative bacteria have been detected and described, including the first report of ESBL incidence in a neutropenic patient at the Port of Spain General Hospital in an isolate of *Salmonella enteritidis*, which exhibited resistance to all penicillins and cephalosporins (including third-generation cephalosporins), aminoglycosides, and trimethoprim-sulphamethazole [3–8]. Several worldwide reports of high prevalence rates and outbreaks of ESBL-producing microorganisms underscore the importance of searching for and detecting their occurrence in all hospitals offering ambulatory services.

The ESBLs are derived from genes for the narrow spectrum beta-lactamases. The most common ESBL types include TEM (named Temoniera in Greece), SHV (sulphydral variable), and CTX-M (reference to its preferential hydrolytic activity against cefotaxime, CTX as its acronym, M for Munich). However, there are others that include the OXA type and some even more uncommon enzymes such as Belgium (BEL-1), *Pseudomonas* extended resistance (PER), Brazil (BES-I), Vietnam extended spectrum beta-lactamase (VEB), and *Serratia fonticola* I (SFO-1) [9]. The majority of the ESBLs that are detected are either of the SHV or TEM types and sometimes result in infections caused by Gram-negative organisms. These infections are sometimes nosocomial by the organisms that produce these enzymes [9]. In a study by Tillekeratne et al., infections caused by ESBL-producing organisms were most frequently diagnosed in outpatients [10]. However, in the study conducted by Fernando et al., ESBL-producing organisms, particularly *Klebsiella* species, seem to be responsible for most nosocomial cases of urinary tract infections [11]. Other ESBL-producing *Enterobacteriaceae* have been described in a study conducted by Briongos-Figuero to cause urinary tract infections [12], while other infections such as bacteremia, intra-abdominal infections, and respiratory tract infections have been described by Pilmis et al. [13].

Some risk factors are associated with the development of ESBL-producing organisms that cause urinary tract infections (UTIs). These include the previous use of antibiotics, history of urinary tract infections, previous hospitalization and duration, previous uncontrolled catheterization, age (occurs mostly in older people), enlarged prostate in males, congenital abnormalities, renal stones, and diseases such as diabetes mellitus [14]. To prevent the spread, especially in hospitals, healthcare workers need to wash their hands regularly as the contaminated health care worker is a frequent mode of transfer between patients [9]. Environmental cleaning is vital and instruments used on patients such as thermometers and blood pressure cuffs should be disinfected frequently as this will aid in the reduction of the spread of these organisms, which will have a positive effect on the community due to the reduction in the spread of these organisms in the community. Prevention is vital as these organisms are able to cause resistance, particularly to penicillins, broad-spectrum cephalosporins, aztreonam, and carbapenems because they have similar basic molecular structures [15], which could lead to treatment failure. As a result of this, rapid and accurate testing methods are necessary.

## 2. Definition and Classification of ESBL

Beta-lactamases are enzymes that hydrolyze *β*-lactam antibiotics, thereby rendering them ineffective [16]. Hydrolysis means that these enzymes target and cleave the chemical bonds of these antibiotics, resulting in the structural and chemical breakdown of these compounds [15]. Extended spectrum beta-lactamases (ESBLs) are defined as beta-lactamases that can hydrolyze and cause resistance to first-, second-, and third-generation cephalosporins, penicillins, and monobactams like aztreonam. However, ESBLs do not cause resistance to cephamycins, namely, cefoxitin and cefotetan, carbapenems, namely, ertapenem, imipenem, and meropenem, and are inhibited by clavulanic acid [17].

There are two schemes of classification in which beta-lactamases are grouped. These include the one that groups them according to functional similarities (Groups 1 to 4) known as the Bush–Jacoby–Medeiros classification, and the other that groups them according to similarity in amino acids, a molecular classification of four classes A to D, known as the Ambler classification [18, 19]. The presence of a serine radical would classify them as serine beta-lactamases, while the presence of a zinc ion at the active site of the enzyme would classify them as metallo-beta-lactamases [18, 19].

Class B beta-lactamases include metalloenzymes that require zinc ions to enable beta-lactam hydrolysis. The action involves the utilization of zinc ions to destroy the beta-lactam ring of the antibiotics. Enzymes that hydrolyze substrates by way of an active site serine upon the formation of an intermediate such as an acyl enzyme belong to the other three classes, A, C, and D. The mechanism of action of serine beta-lactamases involves the destruction of the beta-lactam ring of the antibiotic using the free hydroxyl group, which is located on the serine residue side chain at the active site of the enzyme, thus resulting in the production of a covalent acyl ester, a process that inactivates the antibiotic [17].

Cephalosporinases are group 1 enzymes belonging to the molecular class C. These enzymes are encoded on most *Enterobacteriaceae* chromosomes. They have more activity against cephalosporins than benzylpenicillin. They also have activity against cephamycins and a few that lack activity on cefoxitin. These enzymes are mostly resistant to clavulanic acid inhibition and show resistance to carbapenems. In some organisms, such as *Pseudomonas aeruginosa, Serratia marcescens,* and *Enterobacter cloacae*, low AmpC (cephalosporinase) expression can be induced when exposed to particular beta-lactams, namely, ampicillin. Amoxicillin, imipenem, and clavulanic acid [20] Group 1 plasmid-mediated enzymes belonging to the families of the Amp type (ACT), cefoxitin (FOX), cephamycin (CMY), and MIR and DHA (both named based on the location where they were discovered, Miriam Hospital in Providence and the Dhahran Hospital in Saudi Arabia, respectively) are also in Group 1 but are not as common as the 2br subgroup of plasmid-mediated ESBLs [20].

Beta-lactamases of the largest group are the functional group 2 serine beta-lactamases, which include the A and D molecular classes. All belong to the molecular class A, except for the 2d subgroup, which belongs to molecular class D. A small group of beta-lactamases prevalent in Gram-positive organisms belong to the subgroup 2a penicillinases. These enzymes have limited the hydrolyzing activity of carbapenems and cephalosporins but hydrolyze penicillins with higher affinity. They are inhibited by clavulanate and tazobactam, with inhibitory concentrations of 50%, usually less than 1 M [18]. Some of these subgroup enzymes are plasmid-encoded, including some Staphylococcal penicillinases, but most are chromosomal.

In another subgroup, the 2b beta-lactamases have high activity of hydrolysis of penicillins and cephalosporins and are inhibited by clavulanate. The enzymes belonging to this group were SHV-1, TEM-1, and TEM-2. However, more TEM and SHV 2b enzymes have been characterized and described in this group [21]. The 2be subgroup represents ESBLs that have a broad spectrum and hydrolyze aztreonam and cephalosporins of the third-generation [9]. The largest of this subgroup evolved by SHV-1, TEM-1, and TEM-2 amino acid substitutions, which achieved a broad spectrum by decreasing the hydrolysis activity of benzylpenicillin. CTX-M enzymes also belong to this subgroup. These enzymes are functionally similar to TEM and SHV. They belong to *the Kluyvera species* and are chromosomally located [18, 22]. These enzymes hydrolyze cefotaxime and are inhibited by tazobactam more than clavulanic acid [21]. Other ESBLS that are not related to CTX-M, SHV, or TEM and are less common also belong to this subgroup. These include the Vietnam extended spectrum beta-lactamase (VEB), Belgium (BEL-1), *Pseudomonas* extended resistance (PER), Brazilian (BES-1), Tlahuicas (TLA-I, TLA-2), and *Serratia fonticola* (SFO-1) [18].

A broad-spectrum group of beta-lactamases that are resistant to clavulanic acid with inhibiting concentrations of 50% or ≥1 M and still have the spectrum of activity of the subgroup 2b enzymes are those of the 2br group. The enzymes that belong to this category include TEM-30, TEM-3, and SHV-10, among many others [23]. Another subgroup, also referred to as complex mutant TEM (CMT) beta-lactamases are the 2ber subgroup. These include TEM enzymes such as TEM-50 [18], which have an extended spectrum, but the inhibition is relative as it relates to clavulanic acid. Subgroup 2e enzymes are penicillinases that are inhibited by clavulanic acid with inhibitory concentrations of 50% <1 M and are able to demonstrate at least 60% hydrolysis of carbenicillin or ticarcillin at the same rate as benzylpenicillin with hydrolysis rates of oxacillin or cloxacillin less than half of that of benzylpenicillin [18]. Another subgroup, 2ce, includes the extended spectrum carbenicillin RTG-4, also termed CARB-10, which has activity against cephalosporins such as cefepime [24].

Subgroup 2d includes OXA enzymes, so called because they are capable of exhibiting hydrolyzing action on oxacillin or cloxacillin at a rate that is 50 percent of that of benzylpenicillin [18]. Most beta-lactamases that belong to this subgroup have inhibitory concentrations of clavulanic acid of 50% greater than or equal to 1 M and are inhibited by sodium chloride. There are subgroups on 2d, including subgroups 2de and 2df. 2de subgroup enzymes have an extended spectrum hydrolyzing activity to oxacillin or cloxacillin and activity to oxyimino beta-lactams such as ceftazidime and cefotaxime, but not to carbapenems. *Pseudomonas aeruginosa* is the organism where this type of beta-lactamase commonly occurs. Most of these enzymes are derivatives of OXA-10 with one and nine amino acid substitutions and include OXA-11 and OXA-15 enzymes. The 2df enzymes, however, mostly occur in *Acinetobacter baumannii* and are mostly chromosomally encoded. These enzymes have the ability to hydrolyze carbapenems.

The 2e cephalosporinases can hydrolyze cephalosporins and are inhibited by clavulanate [9]. The Amp enzymes of group 1 occur in organisms similar to this group of enzymes, and they demonstrate resistance in a similar way, making it difficult to be distinguished. However, a differentiating characteristic is that of low aztreonam affinity on the part of the amp enzymes. The two subgroups included the molecular class A serine carbapenemases. These enzymes are inhibited by tazobactam more profoundly than clavulanic acid, and as the name suggests, the substrates are carbapenems. *Klebsiella pneumoniae* carbapenemases (KPC) belong to this group and have been associated with Gram-negative infections worldwide. The first report in the world was identified in Columbia (in 2006) in a *Pseudomonas aeruginosa* isolate, which produced KPC-2 [25]; the second was in Puerto Rico [26], and the third isolate was reported in Trinidad and Tobago [27].

The metallo-*β*-lactamases (MBLs) belong to group 3 and molecular class B. In terms of the structure, these enzymes require zinc ions at their active sites. In terms of function, they were differentiated based on their ability to hydrolyze carbapenems similar to serine beta-lactamases. However, compared to serine beta-lactamases, they are not inhibited by clavulanate but by ethylene diamine tetra-acetic acid (EDTA) and have a low hydrolyzing ability for monobactams. In clinical isolates, these enzymes are often produced along with a second or third beta-lactamase. In terms of subdivisions, there are three, namely, B1, B2, and B3 based on structure and 3a and 3b based on function [18]. The 3a subgroup includes the imipenase (IMP) and the Verona integron-boring metallo beta-lactamase (VIM), and they belong to the Bl subclass. They require two zinc ions, which are amino acids bound to achieve hydrolyzing activity. *Caulobacter vibriodes* (CAU-1), *Fluoribacter gormanii* (FEZ-1), *Elizabethkingia meningoseptica* (GOB-1), and the L1 metallo-beta-lactamase of *Stenotrophomonas maltophilia* are of this subgroup 3a, and of the B3 subclass. The subgroup 3b enzymes exhibit the hydrolysis of carbapenems effectively once there is occupancy of only one zinc binding site. They have better hydrolyzing activity of carbapenems compared to cephalosporins and penicillins. *Aeromonas hydrophilia* CphA and *Serratia fonticola* Sfh-l enzymes belong to the B2 subclass. Group 4 includes penicillin-hydrolyzing beta-lactamases that are resistant to clavulanic acid inhibition. These enzymes undergo profound hydrolysis to cloxacillin and carbenicillin. These enzymes do not belong to the molecular class [28].

### 2.1. Types of ESBL

#### 2.1.1. SHV

A procedure involving the inhibition of SLV led to the designation of the SHV gene to be a sulfhydryl variable as it was thought that P-chloromercuribenzoate substrate variability and relatedness was due to the substrate that was engaged in the procedure [9]. SHV-1 was first discovered in Switzerland in 1974. SHV-1 beta-lactamases are plasmid-mediated, meaning they are spread by plasmids and occur most frequently in *Klebsiella pneumoniae.* The SHV-1 enzymes have no hydrolytic effect on cephalosporins, which are broad-spectrum agents such as ceftriaxone and ceftazidime; however, they can hydrolyze those with a narrow spectrum, such as cephalothin and cefazolin. They also cannot hydrolyze cephamycins and carbapenems, but can hydrolyze penicillins [9]. The mechanism of action involves hydrolysis, which is achieved by attacking the beta-lactam ring of the antibiotic using the free hydroxyl group, which is located on the serine residue side chain at the active site of the enzyme. The result is the production of a covalent acyl ester, which inactivates the antibiotic. The SHV types that have an ESBL phenotype are described based on serine replacement at position 238 for glycine. These beta-lactamases due to this mutation have extended spectrum characteristics [9].

The SHV-2 enzyme was first isolated in Germany from a *Klebsiella ozaenae* isolate in 1983. This enzyme demonstrated extended spectrum activity against cefotaxime and ceftazidime. The serine residue at position 238 is needed for effective ceftazidime hydrolysis, and a lysine residue is needed for effective cefotaxime hydrolysis. The SHV-3 enzyme was discovered in France from a *Klebsiella pneumoniae* isolate in 1988, and SHV-3 is similar to SHV-2 as they both share extended spectrum activity. A point mutation led to the replacement of leucine at position 205 in SHV-2 with arginine in SHV-3. This changes the isoelectric point from 7.6 (for SHV-2) to 7.0 (SHV-3) [9]. The isoelectric point refers to amino acid migration.

The SHV-4 also has extended spectrum activity similar to SHV-3 and was discovered shortly after in France from a *Klebsiella pneumoniae* isolate. A point mutation caused amino acid replacement at position 240 with lysine for glutamic acid. The isoelectric point was 7.8. SHV-5 was discovered in Chile from a *Klebsiella pneumoniae* isolate, and its replacement was at position 240 with lysine, but with an isoelectric point of 8.2. SHV-6 was also discovered in France in 1991 from a *Klebsiella pneumoniae* isolate. There is a difference with the other SHV types based on its hydrolyzing action against the ceftazidime antibiotic and not against cefotaxime. Both SHV-1 and SHV-6 differ at position 179, where there is a replacement of aspartic acid in SHV-1 with alanine of SHV-6. The same isoelectric point of 7.6 is common to SHV-2 and SHV-6, which emphasizes the importance of accurate and rapid methods to distinguish between these enzymes [29–31].

The SHV enzymes have also been identified in other organisms. For example, SHV-7 was first identified in *Escherichia coli* in the United States of America. It has an isoelectric point of 7.6, which is caused by amino acid substitution. There was a replacement of isoleucine at position 8 of SHV-5 with phenylalanine of SHV-7 and a substitution of serine in SHV-7 at position 43 with arginine [32]. SHV-8, also discovered in the United States of America, has an isoelectric point of 7.6. This enzyme has a replacement of asparagine with aspartate at position 179 [33].

The SHV-9 was discovered in Greece from three organisms: *Escherichia coli*, *Serratia marcescens*, and *Klebsiella pneumoniae* in 1995. Here, there is a replacement of alanine at position 140 with arginine [34]. Most SHV variants have the ESBL phenotype, although there is one SHV variant that has an inhibitor-resistant phenotype, SHV-10 [35]. This enzyme is a derivative of SHV-5, which has an extra amino acid replacement at serine 130 with glycine. SHV-11 and SHV-12 were discovered in Switzerland with SHV-11 lacking extended spectrum activity against cephalosporins. Both enzymes have a replacement of leucine at position 35 to glutamine with SHV-11 derived from an SHV-1point mutation and SHV-12 from an SHV-5point mutation [35]. To detect these mutations, polymerase chain reaction single-stranded conformation polymorphism (PCR-SSCP) may be used, which involves the use of a very short primer; the strands of the deoxyribonucleic acid (DNA) are then separated and then analyzed by electrophoresis. This technique is a rapid method used for point mutation characterization in short DNA sequences and is used in the characterization of SHV genes because point mutations are involved in phenotypic resistance expression. Other genetic methods of detection include molecular techniques that specifically detect a particular gene, such as SHV, which causes ESBL production [36].

Although detected in Switzerland, Germany, France, Chile, and the USA, SHV genes have also been detected in Trinidad and Tobago among *Klebsiella pneumoniae* and *Escherichia coli* isolates; 34.5% SHV was detected in *Klebsiella pneumoniae* isolates and 4.1% in *Escherichia coli* isolates [3]. In Trinidad and Tobago, more recently, 84.8% SHV was reported in *Klebsiella pneumoniae* isolates [5] and 7.8% in *Escherichia coli* isolates [4]. The frequency of SHVs has increased in the country. Extended spectrum SHV beta-lactamases, although found mostly in *Klebsiella pneumoniae* and other Gram-negative bacteria, are now regarded as ESBL with the highest prevalence. SHV enzymes have been observed in nosocomial pathogens such as *Pseudomonas aeruginosa*, although they are mostly observed in *Klebsiella pneumoniae* and *Escherichia coli*. These organisms cause many infections of the urinary tract, abdomen, wounds, bacteremia, respiratory tract, and sepsis [37].

These broad-spectrum SHV enzymes are encoded in plasmids that are mobile, multiresistant, and very transmissible. As a result, they are able to spread to nearly all *Enterobacteriaceae species*. Appropriate treatment is therefore crucial as the presence of these genes that cause ESBL production results in multidrug resistance and consequent treatment failure.

#### 2.1.2. TEM

In the 1960s in Greece, the TEM-1 enzyme was first discovered in *Escherichia coli* isolated from a patient named Temoneira. TEM enzymes are plasmid-mediated and are capable of transferring different Gram-negative bacterial species. These enzymes attack and damage the beta-lactam ring of antimicrobial agents, causing them to lose their function. It has the ability to hydrolyze ampicillin more profoundly than oxacillin and carbenicillin and is inhibited by clavulanic acid [9]. The increasing resistance towards penicillin and ampicillin in *Neisseria gonorrhoea* and *Haemophilus influenzae* is due to this enzyme's capability of hydrolysis.

The TEM-2 was derived from TEM-1, both of which have similar biochemical features, and the difference is in their isoelectric point change (5.4 to 5.6) [9]. These two enzymes hydrolyze cephalosporins, which have a narrow spectrum of activities, such as cephalothin and cefazolin. Cephalosporins of higher generation, which possess an oxyimino side chain that includes ceftriaxone, cefepime, cefotaxime, and ceftazidime, are resistant to their activity [38]. The TEM-3, formerly known as CTX-1, due to its profound activity on cefotaxime, is differentiated from TEM-2 based on two amino acid substitutions. The TEM-3 was considered the first TEM-type to be termed extended-spectrum beta-lactamase (ESBL) [39, 40]. There are more than 100 other TEM derivatives that are described based on amino acid sequences, including SHV beta-lactamases and OXA [41]. Most of these derivatives are considered ESBLs. Enzymes with low susceptibility to beta-lactamase inhibitors and no hydrolyzing action on broad-spectrum cephalosporins are not considered ESBLs. The active site of the enzyme has an ESBL phenotype cluster around it, which is due to the amino acid substitutions. This substitution caused configurational changes, making it accessible to oxyimino beta-lactam substrates, and as a result, increases the susceptibility to inhibitors such as clavulanic acid. Usually, ESBL with a broad-spectrum contains more substitutions than a single amino acid [42].

Although *Klebsiella pneumoniae* and *Escherichia coli* are the major organisms where TEM-type ESBLs are most frequently detected, in Italy, another TEM-type beta-lactamase was detected in an isolate of *Serratia marcescens* [43]. This enzyme is termed TEM-AQ. There are replacements of amino acids that were not observed in other TEM types [32]. There was a large outbreak in the country of North America caused by TEM-26; at later times, other groups were identified [9]. In Trinidad and Tobago, among *Klebsiella pneumoniae* and *Escherichia coli*, 100% TEM was identified in *Escherichia coli* isolates and 84.3% in *Klebsiella pneumoniae* isolates [6]. More recently, in Trinidad and Tobago, 59% TEM was detected in *Klebsiella pneumoniae* isolates [8] and 13.3% in *Escherichia coli* isolates [7]. It is important to rapidly detect infections with bacteria carrying the specific TEM beta-lactamase gene to determine the appropriate treatment. The determination of the presence and spread of these TEM genes is important for understanding their epidemiological profiles. This can be done by molecular techniques, as discussed later.

#### 2.1.3. CTX-M

The CTX-M enzymes are functionally similar to TEM and SHV enzymes and belong to beta-lactamases in *Kluyvera species*, which are chromosomally determined [18, 22]. These enzymes target and cleave the chemical bond in the beta-lactam ring of beta-lactam antibiotics, rendering these antibiotics ineffective against bacteria.

In Germany, CTX-M-1 was identified in an E. *coli* isolate in 1989. It was named because of its profound ability to hydrolyze cefotaxime. It was also found in France in an *Escherichia coli* organism in 1992 and was termed MEN-1 [9]. Similar to MEN-1, in Japan, in 1993, another enzyme was isolated from an *Escherichia coli i*solate that was, however, resistant to cefotaxime; this was named as Toho-1, after the location where this case occurred, at the Toho hospital in Tokyo. Subsequently, it was renamed CTX-M-2. Other CTX-M types have been detected in China from other *Enterobacteriaceae* isolates. Many other CTX-M enzymes have also been detected in Trinidad and Tobago. A novel CTX-M-2 enzyme was detected in *Klebsiella pneumoniae* in patients treated in a hospital located in the country [6], and 58.8% CTX-M occurrence was also reported in the country detected in *Escherichia coli* [7] and 46.9% of both CTX-M types 1 and 2 from *Klebsiella pneumoniae* isolates [8]. There was a large outbreak in 2000–2002 in Calgary, Canada, in which there were infections caused by organisms, including *Klebsiella pneumoniae* that produced CTX-M-14 [44, 45]. Several infections, including those of the bloodstream and urinary tract, are caused by *Escherichia coli* and, in particular, strains that produce type 15 CTX-M [9].

The CTX-M is a plasmid-encoded plasmid carrying resistance genes for other antibiotics such as tetracycline, aminoglycosides, and sulfamethoxazole/trimethoprim. These enzymes can hydrolyze other cephalosporins (cefotaxime, cefepime, and ceftazidime) and hydrolyze aztreonam [46]. However, they were inhibited by clavulanic acid. To appropriately treat infections resulting from ESBL-producing organisms caused by these enzymes, the microbiology laboratory should be capable of accurately detecting these ESBL-producing organisms. Failure to do this may lead to outbreak of multidrug-resistant pathogens and treatment failure. Phenotypically, ceftazidime resistance is used to indicate the presence of ESBL such as CTX-M; however, this method is not reliable, as some isolates may not be susceptible to this antibiotic [9].

#### 2.1.4. OXA

The OXA types are ESBLs, which have characteristics different from those of TEM and SHV enzymes belonging to the functional group 2d and molecular class D [18, 47]. The name OXA was designated because of its ability to hydrolyze oxacillin. These enzymes are resistant to cephalothin and ampicillin. Another main characteristic is the profound hydrolyzing ability of oxacillin and cloxacillin to be greater than 50 percent of that of benzylpenicillin and with a poor clavulanic acid inhibition [18]. Belonging to class D indicates that it is a serine beta-lactamase. The mechanism of action of serine beta-lactamases is by attacking the beta-lactam ring of the antibiotic using the free hydroxyl, thereby resulting in a covalent acyl ester, thus damaging the antibiotic and causing it to be inactive. Most of these OXA enzymes are not termed ESBLs because of their lack of hydrolyzing capabilities. However, early OXA beta-lactamases were differentiated based on isoelectric points and their ability to be inhibited by chloride ions, OXA-1 and OXA-2 were differentiated from OXA-3 based on this inhibition feature, as both OXA-I and OXA-2 were by these ions. However, OXA-10, which is referred to as ESBL, weakly hydrolyzes aztreonam, ceftriaxone, and cefotaxime. There are others, such as OXA-45, -35, -32, -31, -28, -19, -18, -17, -16, -15, -14, and -11, which have stronger resistance to these antibiotics [48].

*Pseudomonas aeruginosa* is the organism where this OXA type of beta-lactamase commonly occurs, and the enzyme was first discovered in an isolate of this organism in Turkey in 1991 [49]; it was multiresistant, including ceftazidime and was found to have amino acid replacement at position 143 of serine to arginine and at position 157 of glycine to aspartate. The hydrolytic activity against ceftazidime increased as a result of this mutation. This mutation also led to OXA-10 derivatives, including OXA-11, -17, -16, -19, -28, -13, and -14, all of which have been detected in *Pseudomonas aeruginosa* [50]. OXA-11, at position 143, has an extra mutation leading to asparagine replaced with serine, and OXA-16 at position 127 also has an additional mutation with replacement of alanine with threonine. Compared to OXA-10, there are nine mutations in OXA-19; OXA-17 at position 73 has a replacement of asparagine with serine. Derivatives such as OXA-15, at position 149, have a replacement of aspartate with glycine, and OXA-36 at position 149 has aspartate replaced with tyrosine. The OXA-18, not derived from but closely related to OXA-19, is inhibited by clavulanic acid, although a characteristic of OXA enzymes is the lack of inhibition [51, 52]. OXA-23 was found in the United Kingdom in an *Acinetobacter baumannii* isolate in 1985 [53]. This enzyme and its derivatives, OXA-27 and OXA-146, have hydrolytic activity against carbapenems, aztreonam, oxacillin, and oxyimino cephalosporins. OXA-146, unlike others, is capable of hydrolyzing ceftazidime (an extended-spectrum cephalosporin) and is the first carbapenemase to demonstrate ESBL characteristics [54]. OXA-40 was detected in Spain from *Acinetobacter baumannii* in 1997, and OXA-40 like genes have also been detected in plasmids in *Klebsiella pneumoniae* and *Pseudomonas aeruginosa*. OXA-40 derivatives are OXA-26 and OXA-25 [55]. These enzymes do not possess high activity against carbapenems and cephalosporins but are able to hydrolyze penicillins, and OXA-51 was detected in *Argentina* from *Acinetobacter baumannii* in 1996, which is chromosomally encoded. OXA-51 has a low hydrolyzing action against carbapenems [56]. OXA-58 was detected in France in *Acinetobacter baumannii* in 2003. These enzymes are able to hydrolyze cephalothin but are unable to hydrolyze cefepime, ceftazidime, or cefotaxime. These enzymes have low hydrolyzing activity against penicillin and carbapenems. OXA-58 variants are OXA-96 and OXA-97, both of which have been identified in *Acinetobacter baumannii*, OXA-96 in 1996 in Singapore and OXA-97 in Tunisia in 2001–2005 [55–57]. OXA-48 was detected in Turkey from *Klebsiella pneumoniae*, which was isolated in 2001. This was multidrug resistant. Resistance included carbapenems. OXA-48 was spread to other Gram-negative isolates. Most OXA enzymes are plasmid-encoded, which are self-transmissible and able to spread between different bacterial species. The OXA enzyme OXA-48, which has been found in *Enterobacteriaceae* with carbapenem resistance, is concerning. Carbapenem resistance among these OXA enzymes coupled with their transferability is a major problem concerning the clinical efficacy of these antibiotics. Carbapenems are important antibiotics that are mainly used for the treatment of severe infections caused by Gram-negative organisms [58–60].

#### 2.1.5. ESBLs

There are some uncommon ESBLs that are plasmid-encoded, found mainly in *Pseudomonas aeruginosa,* namely, the PER-*Pseudomonas* extended resistance beta-lactamase [61], integron-located VEB-1 extended spectrum beta-lactamase [62], and the GES-Guiana extended spectrum beta-lactamase [63]. There are others that have been found in *Enterobacteriaceae*, such as SFO-1-*Serratia fonticola* 1 [64]. The ESBLs of the PER type have mostly been found in Turkey. They share approximately 25% homology with other ESBLS, TEMs, and SHVs. PER-1, found in *Pseudomonas aeruginosa* and later in *Acinetobacter* species [65], and *Salmonella enterica* serovar Typhimurium are able to hydrolyze *β*-lactam antibiotics [66]. It was inhibited by clavulanate. PER-1 was also found in Italy from *Pseudomonas aeruginosa* and *Proteus mirabilis* and in Korea from *Acinetobacter* species. PER-2 has been detected in both *Klebsiella pneumoniae* and *Escherichia coli* [67].

There was 38% homology between VEB-1, PER-1, and PER-2 ESBLs. This enzyme was found in France in *Escherichia coli* isolated from a Vietnamese patient and was later found in *Pseudomonas aeruginosa* in Thailand. Another VEB enzyme, VEB-3, was found in China from *Enterobacter cloacae* [62]. In Brazil, GES-I has been found in *Pseudomonas aeruginosa* and has also been found in *Klebsiella pneumoniae*. GES-2 has been detected in South Africa from *Pseudomonas aeruginosa* and GES-3 in Japan from *Klebsiella pneumoniae*. GES-14 was detected in France from *Acinetobacter baumannii* and is resistant to penicillins, cephalosporins, and carbapenems [68–70]. IBC-1 was detected in Greece from *Escherichia coli* and *Enterobacter cloacae*, and IBC-2 was also detected in Greece but from *Pseudomonas aeruginosa*. This enzyme is highly resistant to ceftazidime, but not as highly resistant to oxyimino cephalosporins [71, 72]. BES-I was isolated from *Serratia marcescens* in Brazil [73]. TLA-1 was isolated from *Escherichia coli* in Mexico City, Mexico. These enzymes demonstrate resistance to some cephalosporins, especially ceftazidime and aztreonam [74]. SFO-1 is a transferrable enzyme that has been isolated from *Serratia fonticola* and *E. cloacae*. It is inhibited by clavulanic acid and has no hydrolytic activity against cephamycins [75].

Although uncommon, plasmid-mediated, these plasmids carry genes of antibiotic resistance and are transferrable between bacteria. Therefore, rapid and accurate methods of detection capable of characterizing these genes are recommended to provide results with specificity and sensitivity in a timely manner to achieve efficient treatment for infected persons. Such methods include PCR assays for the detection of ESBLs.

## 3. Treatment of ESBL Infections

Being the most commonly plasmid encoded, ESBLs are able to confer resistance to penicillins, cephalosporins, and aztreonam by hydrolyzing these antibiotics [9]. Cephalosporins are widely used in hospitals to treat many infections, including urinary tract infections. There might be a poor outcome if an infected patient with ESBL producers is treated with an antibiotic to which there is resistance. In a study, risk factors for colonization due to carbapenem-resistant ESBL-producing *Enterobacteriaceae* including lengthy stays in health care facilities were examined [76]. Carbapenems were the most effective against ESBL-producing bacteria *in vitro.* Third-generation cephalosporins do not exhibit very good activity against ESBL-producing organisms. Carbapenem-resistant *Enterobacteriaceae* (CRE) were listed as an urgent threat in a report from the Centers for Disease Control describing the top 18 drug-resistant threats, and nearly half of the patients hospitalized with bloodstream infections caused by CRE died. Medical personnel rely on clinical expertise and secondary data sources to assist clinicians with treatment options for UTIs and other infections. Rapid and accurate testing methods to detect and monitor resistant genes mediating ESBL are required to assist clinicians in the management of these infections. Antibiotics are available for the treatment of various types of infections. Sites of infection, such as urinary tract infection or infections of the blood, require appropriate antibiotics for treatment.

Appropriate treatment is important as ESBL-producing organisms pose a great threat to health care delivery because of multidrug resistance and treatment failure [9]. Quinolones may be used as the main treatment option for patients with complicated UTIs caused by ESBL producers. Other recommended treatments for ESBL infections include carbapenems, which may be used as therapy for treating bacteremia, hospital-acquired pneumonia, and intra-abdominal infection and quinolones as second-line therapy if the organism is susceptible. In the case of meningitis, meropenem is recommended as the therapy of choice and intrathecal polymyxin B as second-line therapy [9]. Furthermore, fosfomycin, nitrofurantoin, and pivmecillinam are important oral treatment options for urinary tract infections caused by ESBL-producing organisms.

## 4. Methods of Infection Control of ESBL Infections

Epidemiologically linked nosocomial infections may occur as epidemics (outbreaks) or as endemic (regular occurrences) [77—79]. It is important to determine whether nosocomial infections are monoclonal or polyclonal. If monoclonal (infection caused by the same clone of organisms), the organisms are spread by way of some method between patients, and if polyclonal, infections are possibly caused by selective pressure inferred using antibiotics [9]. According to a study, most people infected with ESBL-producing bacteria had been hospitalized for an average of 11 to 64 days prior to developing an infection [79].

To prevent the spread of ESBL-producing organisms within hospitals, healthcare workers need to wash their hands regularly, as this is the most common mode of transfer from patient to patient [80]. Environmental cleaning is vital and instruments used on patients, such as thermometers and blood pressure cuffs, should be disinfected frequently, as this will limit the spread of these organisms. When these organisms are already endemic, these methods should be followed: (a) infected patients should be identified with the use of appropriate laboratory testing methods as an infection control measure, (b) rectal swabs should be collected and cultured on appropriate media to identify those patients who have been colonized, (c) molecular epidemiology testing of organisms from both the infected and those colonized should be performed, (d) isolation procedures to avoid contact with infected persons and if many types of strains are demonstrated to implement controls on antibiotic use should be implemented [6]. If not endemic, the following measures should be adhered to: (i) regarding persons who have been infected or colonized, isolation of such persons should be done, (ii) the source of infection from the environment should be detected and monitored, and (iii) rectal swabs should be taken to detect the presence of ESBL production in people who have been colonized but not infected [9].

## 5. Detection Methods for ESBL

**Figure 1:**
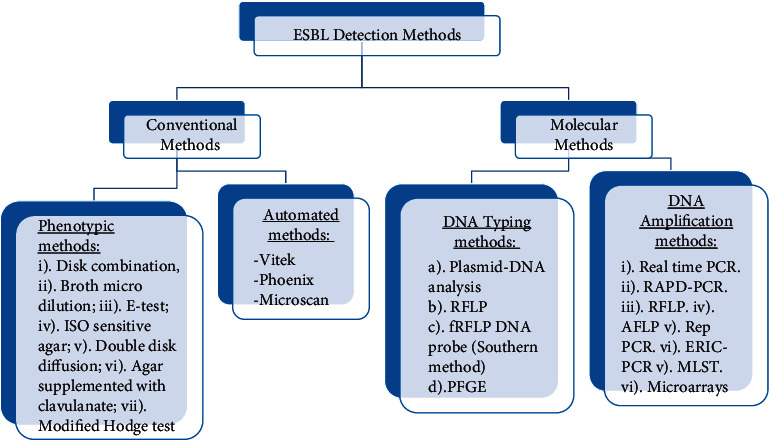
Simple flow chart of extended spectrum beta-lactamase enzyme detection in any microbiology laboratory. ESBL = extended spectrum beta-lactamase; DNA = deoxyribonucleic acid; RFLP = restriction fragment length polymorphism; fRFLP; PFGE = pulsed field gel electrophoresis; PCR = polymerase chain reaction; RAPD-PCR = random amplification of polymorphic-PCR; RFLP-PCR = restriction fragment length polymorphism-PCR; AFLP = amplified fragment length polymorphism; Rep-PCR = repetitive extragenic palindromic-PCR; ERIC-PCR = enterobacterial repetitive intergenic consensus-PCR; MLST = multilocus sequence typing.

There are several methods of detecting ESBL in any microbiology laboratory as described below, and the flow chart in Figure 1 gives a summary of the methods.

### 5.1. Conventional Detection Methods

#### 5.1.1. Screening Methods

The disk diffusion-recommended methods are based on guidelines from the Clinical and Laboratory Standards Institute (CLSI) based on the specific zone diameters of the recommended antibiotic disks to screen for ESBL production [81].

The dilution method: again, this is based on CLSI guidelines, depending on which organism is being tested for possible ESBL production. MIC values >21 g/mL suggest ESBL production. However, screening for cefpodoxime with an MIC of ≥8 pg/ml suggests ESBL production [81], including specific screening plates (e.g., KPC/C3G biplates).

#### 5.1.2. Phenotypic Confirmation Tests for ESBL

(1) Combination disks. Again, this is based on CLSI guidelines. Cefotaxime or ceftazidime (with clavulanic acid present or absent) were used as disks. Here, Mueller–Hinton agar was used and phenotypic confirmation was made by zone diameter detection between the disks employed [81].

(2) Broth microdilution. This method was performed according to standards with the use of cefotaxime in addition to clavulanate (0.25/4 to 64/4 *µ*g/ml). Cefotaxime alone (0.25 64 g/ml), and ceftazidime in addition to clavulanic acid up to 128 mg/mL, and ceftazidime by itself up to 128 mg/mL [81]. were used. A greater than or equal to three–twofold serial dilution reduction in the MIC forms the basis of phenotypic confirmation testing. There are recommendations for the use of ATCC 25922 *E. coli* and ATCC 700603 *K. pneumoniae*, which represent a negative control and positive control in ESBL production screening and phenotypic testing.

#### 5.1.3. Automated Methods for ESBL Production

There are several automated methods, including Vitek (Biomerieux), Phoenix (BD Diagnostics), and Microscan (Siemens), which are used to screen or confirm ESBL production. Vitek employs cefotaxime, ceftazidime, and clavulanic acid [82].

A positive ESBL result is based on observing the wells for growth levels. There is an incubation period after which a positive result is determined by reduced growth in the ceftazidime well containing clavulanic acid, and a comparison was made with the well containing cephalosporin only with respect to growth. Vitek 2 was found to perform reliably with CLSI breakpoints [83]. The specificity and sensitivity of this automated system exceeded 90% [83, 84]. The results are available in 4–18 h. The Phoenix automated system utilizes the detection of clavulanic acid in combination with cefotaxime, ceftazidime, and ceftriaxone antibiotics for the detection of ESBL production. MIC results were available in 6–16 h. The Microscan system involves manual inoculation of hydrated microdilution trays, which are then placed on the machine in the incubator slots. In this system, growth is determined by using a fluorometer. The performance of this system was evaluated and was found to yield 91% performance reliability [85]. The results are available from four to 18 h.

#### 5.1.4. E-Test

These strips were used for the screening and phenotypic confirmation of ESBL production. One end of the strip contained clavulanic acid plus ceftazidime, while the other end contained only ceftazidime. Strips containing cefotaxime, instead of ceftazidime, may also be used. The MIC was determined according to the manufacturer's guidelines. The E-test has reported up to 95% sensitivity and specificity [86].

#### 5.1.5. ISO-Sensitest Agar

This method is based on a ratio calculation where the division of the combination disk zone size by the cephalosporin zone size and ESBL production is suggested if there is a result upon completing this process of division of greater than 1.5 [87].

#### 5.1.6. Double Disk Diffusion Test

This method utilizes the synergy between cefotaxime and clavulanic acid by placing an amoxicillin/clavulanic acid (20 *μ*g/10 g) disk and a cefotaxime (30 *μ*g) 30 mm apart. The presence of ESBL is indicated by an extension of the cefotaxime inhibition zone toward the disk containing clavulanic acid [82].

#### 5.1.7. Agar Supplemented with Clavulanate

This method utilizes a comparison of zone sizes to determine the presence of ESBL production using supplemented Mueller–Hinton agar with 4 mg/mL of clavulanic acid and unsupplemented Mueller–Hinton agar. Ceftazidime, ceftriaxone, and aztreonam (all of 30 g) were placed on both plates, and ESBL production was then interpreted by differences in zone diameters [88].

#### 5.1.8. Modified Hodge Test (MHT)

This test was used to detect the carbapenemases. A carbapenem disk (meropenem or ertapenem 10 *μ*g) was placed in the center of an inoculated agar plate (Mueller–Hinton) with ATCC 25922 control (*Escherichia coli*). The test organism was streaked from the carbapenem disk to the edge of the plate. Following incubation, a clover leaf appearance of *Escherichia coli* ATCC 25922 growing along the growth streak of the test organism indicated a positive result [89].

### 5.2. Molecular Detection Methods

#### 5.2.1. Typing Methods—DNA-Based

*(1) Plasmid DNA Analysis*. Plasmid DNA analysis is important for evaluating the potential spread of resistant genes, which is epidemiologically important. There is an advantage of a rapid turnaround time of approximately 24 h. Owing to the transferring ability of mobile elements, there is a disadvantage of applying this method to epidemiological studies. Small plasmids, as opposed to larger ones, are suitable for typing because of their stability. There has, however, been a replicon typing, which is PCR-based and targets plasmid replicons, which are useful in plasmid epidemiology [90].

*(2) Restriction Fragment Length Polymorphism (RFLP)*. This method detects fragments of different lengths after the digestion of particular DNA samples. Gel electrophoresis separation was performed, where a DNA fingerprint was visualized in the form of bands. This method is useful in the interpretation of different profiles, for example, in bacteria where there might be a larger number of DNA fragments that pose difficulties in interpretation using standard gel electrophoresis. For easier interpretation, two approaches have been developed, namely, RFLP analysis with the use of a DNA probe and pulsed field gel electrophoresis (PFGE) [91]. The latter has been extensively used in studies from Trinidad and Tobago [92].

*(3) fRFLP Analysis by DNA Probe*. This method is also known as Southern analysis. In this method, a limited number of fragments from a genomic digest was selected by Southern blotting and hybridization. The DNA is digested with the use of restriction enzymes. Electrophoresis was performed to separate fragments. Upon separation, these fragments were placed onto a membrane filter, which was then incubated. Incubation occurs with a probe that hybridizes to a particular gene probe [92, 93]. Ribotyping is based on this RFLP method and has a turnaround time of approximately 1–3 days. This technique was used to analyze the ribosomal DNA. Here, only DNA fragments containing ribosomal RNA sequences were selected. Differences in regions associated with the ribosomal RNA gene allow for variations in fragment sizes, which therefore provides distinguishing patterns, thus making differentiation possible [92].

*(4) Pulsed Field Gel Electrophoresis*. In this method, the restriction patterns of the entire genome were analyzed. Cutting enzymes are used to digest chromosomes. These infrequently cutting enzymes recognize rare DNA sequences, thus reducing the number of fragments that are very large. Upon enzymatic digestion, agarose blocks are applied to the wells of the gel and further exposed to an alternating current voltage, where there is a switch in the direction of the electric field controlled by the device. The disadvantages of this method are that it is time-consuming, laborious, and requires skill for the interpretation of results. The turnaround time was approximately 2–3 days. The usefulness of pulsed field gel electrophoresis has been described in epidemiological studies of extended spectrum beta-lactamase producers [94, 95].

#### 5.2.2. Typing Methods—Amplification-Based

*(1) Real Time Polymerase Chain Reaction*. This process involves cycles inclusive of three phases: the first stage, where the double strand is separated at a temperature of approximately 95°C; the second stage, where the primers bind to the template DNA, at lower temperatures of approximately 50–60°C; and the third stage, where the DNA polymerase facilitates polymerization at a temperature of approximately 72°C. The real-time procedure was conducted in a PCR machine also known as a thermal cycler. The cycler has a detector that detects fluorescence when it is committed. In this process, the amplification of the target DNA was monitored during the experiment and not at the end. Data were collected during the exponential growth phase, and while the number of amplicons were generated, the increase in the fluorescent signal was directly proportional to it. The turnaround time was approximately 2 h. There are two methods involved, including the use of fluorescent dyes that intercalate double-stranded DNA and the other involves the use of a DNA probe, which consists of fluorescent reporter-labeled oligonucleotides. The reporter allows detection only after the probe is hybridized with the complementary sequence. This method has been employed for *Enterobacteriaceae* detection [95].

*(2) Random Amplification of Polymorphic DNA (RAPD-PCR)*. This typing method uses short random primers that anneal to the chromosomal DNA sequences. The process occurs at annealing temperatures that are low, allowing mismatches and, as such, can be engaged in the amplification of genomic regions. The products were separated by agarose gel electrophoresis to obtain the banding patterns. This method has the advantage of a short turnaround time of 24 h and requires only a small amount of DNA when processed for bacterial analysis. This method has been applied to ESBL studies [8, 96].

*(3) Restriction Fragment Length Polymorphism-PCR (RFLP-PCR)*. This is a polymerase chain reaction (PCR)-based method in which restriction analysis is carried out on amplicons using specific primers for particular sequences. The PCR amplicons were then cut using restriction enzymes. Upon cutting the amplicon, the gene should have adequate variable sequences. The turnaround time was approximately 24–48 h. This method has been used in the molecular epidemiological studies of bacterial pathogens [96, 97].

**Figure 2:**
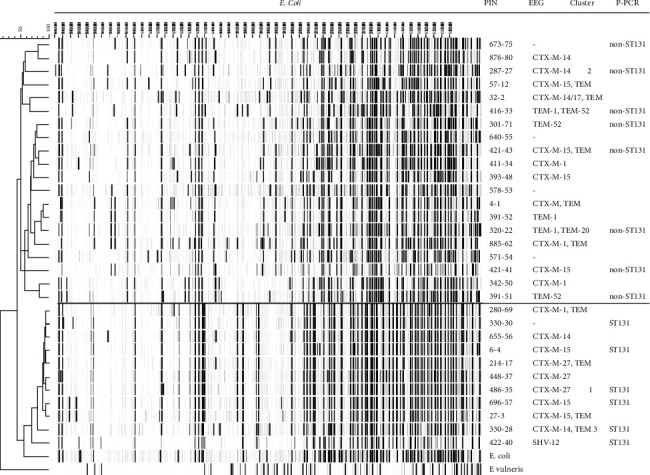
AFLP-results of all *E. coli* isolates with one representative isolate per cluster. Abbreviations: PIN = patient identification number; EEG = ESBL encoding gene; and P-PCR = phylogroup defining polymerase chain reaction. https://doi.org/10.1371/journal.pone.0222200.g001.

*(4) Amplified Fragment Length Polymorphism (AFLP)*. AFLP has two forms. One form involves PCR amplification primers of which there are two primers and two restriction enzymes. The other had one primer and one restriction enzyme. With the use of enzymes, digestion of the whole DNA occurs; then, linkers are ligated to the DNA fragments on which the PCR primers will target. Gel electrophoresis was then used to determine and compare the separation profiles of the amplified fragments. This method involves a large number of steps to be performed during the procedure. However, large numbers of fingerprints can be generated by the use of varying combinations of primers and restriction enzymes. The turnaround time was approximately 40 h [97, 98]. It was reported by van Dulm et al., 2019, that phylogenetic typing on a selection of 19 *E*. *coli* isolates was performed by dividing the phylogenetic tree in half with 49% non-ST131 *E*. *coli* isolates and 51% ST131 *E*. *coli* isolates that are described in Figure 2. Isolates from clusters one and three belong to the ST131 genotype [97].

*(5) Repetitive Extragenic Palindromic PCR (Rep-PCR)*. This method amplifies a variety of repeated bacterial sequences distributed in the genome to produce multiple amplicons of different sizes. One or two primers were used for PCR, followed by gel electrophoresis to achieve amplicon separation. The banding patterns obtained were then compared. This method has the advantage of a short turnaround time of 24 h to obtain results and requires a small amount of DNA for bacterial analysis typing. Factors influencing this method include the conditions of gel electrophoresis, primer selection, and thermal cycling conditions [98–100].

*(6) ERIC-PCR-Enterobacterial Repetitive Intergenic Consensus PCR*. It is also a type of Rep-PCR method that utilizes ERIC sequences. The procedure is a fingerprinting method where targets are located in the DNA space between two genes of a genome and contains 126 base pairs; the turnaround time is 24 h, and has been previously used for *Enterobacteriaceae* gene detection [101, 102].

*(7) Multilocus Sequence Typing (MLST)*. In this method, nucleotide substitutions were compared by analyzing the sequences of multiple genes to determine the genetic relationships among the bacterial strains. Multiple housekeeping genes were mostly sequenced because of their presence in all the isolates. The turn-around time was approximately three days. For the effectiveness of this method in epidemiology, there should be an adequate selection of genes to distinguish appropriately between isolates. Sequence-type relatedness may be determined based on the global optimal eBURST algorithm [103, 104]. Because it requires multiple sequencing reactions, it can be expensive.

*(8) Microarrays*. This technique is based on the generation of cDNA molecules from mRNA, which are labeled, or labeled mRNA molecules, which are hybridized to a specific oligonucleotide probe array. Commercially available assays can be used to identify ESBLs. These include the check-ESBL assay, which utilizes probes that are target-specific and are able to give products when there is a direct match with the target sequence; the microarray hybridization processing time lasts for one to two hours, which depends on the quantity of samples for analysis on each microarray, and a complete turnaround time, with the addition of the amplification process, is about six hours. The check-ESBL allows for the differentiation of narrow-spectrum TEM and SHV from the variants. The check-MDR CT 101 assay targets the same genes in addition to CTX-M; however, it does not target narrow spectrum enzymes [104]. Other studies have been conducted in Trinidad and Tobago on the detection of extended spectrum beta-lactamase (ESBL) and have been extensively published [6, 105, 106].

## 6. Guideline Recommendation for Treatment of ESBL Infections

Antimicrobial-resistant infections are commonly encountered in US hospitals and result in significant morbidity and mortality. A guidance was developed to provide recommendations for the treatment of infections caused by extended-spectrum *β*-lactamase-producing *Enterobacteriaceae* (ESBL-E), carbapenem-resistant *Enterobacteriaceae* (CRE), and *Pseudomonas aeruginosa* with difficult-to-treat resistance (DTR-*P. aeruginosa*). The field of antimicrobial resistance is very dynamic and rapidly evolving, and the treatment of antimicrobial-resistant infections will continue to pose challenges to clinicians. This guidance document is current as of September 17, 2020. The most current version of the guidance including the date of publication can be found at http://www.idsociety.org/practice-guideline/amr-guidance/ [107].

In a recent document, doxycycline was not recommended for the treatment of extended spectrum beta-lactamase *Enterobacteriaceae* (ESBL-E) cystitis due to limited urinary excretion; however, it was not supported by some authors as approximately 35%–60% of an oral dose of 100 mg of doxycycline is excreted unchanged into the urine [108]. Another work reported by Jomehzadeh et al. demonstrated that enteropathogenic *Escherichia coli* (EPEC), which is one of the most important diarrheagenic agents among infants under 5 years in developing countries, is in relation with the distribution of class 1 integrons and ESBLs in highly prevalent EPEC strains. Moreover, the results revealed that continuous monitoring of the emergence and expansion of MDR in EPEC strains is necessary to decrease the incidence of infectious disease [109].

Tanimoto et al. reported five novel *S. fonticola* isolates producing FONA as part of the surveillance for ESBL-producing *Enterobacteriaceae*. The isolates were obtained from a total of 176 samples of imported chicken meat at several quarantines in Japan in 2017 and 2018; four strains were detected in 86 chicken meat samples in 2017 and one was identified in 90 chicken meat samples in 2018. In this procedure, ESBL-producing *Enterobacteriaceae* was first isolated by the selection for cefotaxime (CTX) or ceftazidime (CAZ) resistance (each at 1 mg/L) on deoxycholate-hydrogen-sulfide-lactose agar. Resistant isolates were purified to determine the minimal inhibitory concentrations (MICs) of CTX and CAZ with or without clavulanic acid (CLA). The isolated strains with reduced resistance (1/8 or less of the MIC of CTX or CAZ) in the presence of CLA were further examined for the detection of the ESBL genes blaTEM, blaSHV, and blaCTX-M by multiplex PCR [110, 111].

The last study that we looked at aimed at assessing the prevalence and associated risk factors for extended-spectrum *β*-lactamase (ESBL)-producing *Escherichia coli* and *Klebsiella pneumoniae* (ESBL-EK) acquisition among patients in ICU in Northern Thailand. Overall, the ESBL-EK acquisition rate among patients during the ICU stay was 29.6%. Multivariate logistic regression analysis identified the use of third-generation cephalosporin (*p*=0.008) as a risk factor for ESBL-EK acquisition [112].

## 7. Conclusion

It is important to note that there are several limitations in the use of molecular methods for detection of ESBL in isolates in several developing countries such as high costs of instruments and the need of highly skilled staff with expertise in proteomics and genomics. However, despite these limitations, in the last three to five decades, ESBL detection and occurrence has evolved significantly. From the time the first ESBL was detected in Trinidad and Tobago among *Salmonella* species, great strides continue to be made in their detection among the Gram-negative organisms. Some areas have been covered where *E. coli* and *K. pneumoniae* are involved. There remains a lot to be done. It is hoped that in the near future, greater strides would be made from this humble beginning to detect and report several ESBL-producing organisms in the country. Each laboratory must always use what works for them best in terms of turnaround time, cost, and efficiency of methods, since there may not be one method that fits for all situations. In addition, in this country, we have successfully combined several methods simply because of availability. It is hearty to note that the country has not been facing any dilemma in the treatment or control of the spread of these organisms that are ESBL producers. It will be great to maintain that status, quo as knowledge of their existence continues to unfold regularly.
